# Chikungunya Virus Infection in Blood Donors and Patients During Outbreak, Mandalay, Myanmar, 2019

**DOI:** 10.3201/eid2611.201824

**Published:** 2020-11

**Authors:** Aung Kyaw Kyaw, Mya Myat Ngwe Tun, Takeshi Nabeshima, Aung Min Soe, Thida Thida, Thet Htoo Aung, Thein Thein Htwe, Su Su Myaing, Tu Tu Mar, Thida Aung, Khin Moh Moh Win, Khin Mar Myint, Ei Phyu Lwin, Hlaing Myat Thu, Corazon C Buerano, Kyaw Zin Thant, Kouichi Morita

**Affiliations:** Department of Medical Research, Pyin Oo Lwin, Myanmar (A.K. Kyaw, A.M. Soe, Thida, T.H. Aung, T.T. Htwe. S.S. Myaing, T.T. Mar, T. Aung, K.M.M. Win, H.M. Thu, K.Z. Thant);; Nagasaki University, Nagasaki, Japan (M.M. Ngwe Tun, T. Nabeshima, A.M. Soe, K. Morita);; Mandalay General Hospital, Mandalay, Myanmar (K.M.M. Win);; 550-Bedded Mandalay Children Hospital, Mandalay, Myanmar (E.P. Lwin, K.M. Myint);; St. Luke’s Medical Center, Quezon City, the Philippines (C.C. Buerano)

**Keywords:** chikungunya, chikungunya virus, CHIKV, viruses, blood donors, molecular epidemiology, neurologic manifestations, acute febrile illness, outbreak, zoonoses, Myanmar

## Abstract

In 2019, an outbreak of chikungunya virus infection occurred in Mandalay, Myanmar, and 3.2% of blood donors and 20.5% of patients who were children were confirmed as being infected. The prevalence rate was up to 6.3% among blood donors. The East Central/South African genotype was predominantly circulating during this outbreak.

Chikungunya (CHIK) is an emerging tropical disease caused by CHIK virus (CHIKV; family *Togaviridae*, genus *Alphavirus*), of which there are 3 genotypes: Asian, East/Central/South African (ECSA), and West African (*1*). This virus was detected in Asia during 1954 and has been observed to continuously circulate in countries in southern and Southeast Asia (*2*–*5*). In Myanmar, the first case of CHIKV infection was confirmed serologically in 1973 (*6*), and the CHIKV ECSA genotype was observed in patients during 2010 (*7*).

The signs and symptoms (e.g., fever, rash, and severe joint pain) caused by CHIKV are similar to those caused by dengue virus. Although deaths from CHIK are rare, this disease can cause neurologic manifestations (*8*). The asymptomatic infection rate for CHIKV is 10%–25%. There is a risk for CHIKV transmission by blood transfusion (*9*), but no transfusion-transmitted infections have been documented. In many countries, including Myanmar, CHIKV screening is not routinely performed for blood donors.

We aimed to determine the proportion of blood donors who had CHIKV IgM and CHIKV RNA. Because our study was conducted during the outbreak of CHIKV infection in Myanmar in 2019, proportions of virus-positive children with acute febrile illness during routine dengue surveillance were also determined. We also sought to identify the CHIKV genotype for CHIKV RNA-positive samples.

## The Study

An outbreak of CHIKV infection occurred in Mandalay, Myanmar, during 2019. A cross-sectional descriptive study was conducted among blood donors at the Blood Bank of Mandalay General Hospital and patients at the Mandalay Children Hospital. A total of 500 blood donors and 151 patients who had acute febrile illness but were negative for dengue virus nonstructural protein 1 (NS1) were enrolled. Approximately 20–25 blood donors/week were enrolled, and their blood samples were collected once per week by using saturation method during the peak season of arboviral infection (June–September). Children (<13 years of age) with acute febrile illness were also enrolled.

Blood samples (2 mL) from blood donors and patients in the acute phase of disease were collected after hospital admission. Individual serum samples were tested by using the QuickProfile Chikungunya IgG/IgM Combo Rapid Diagnostic Test Kit (LumiQuick, https://lumiquick.co). IgM-positive samples were confirmed by using an in-house CHIKV IgM capture ELISA (*7*).

Viral RNA was extracted from pooled serum samples (15–20 samples/pool) of blood donors and from individual samples of patients by using the Viral RNA Extraction Kit (QIAGEN, https://www.qiagen.com). Conventional 1-step RT-PCR was performed by using the Invitrogen 1-step RT-PCR Kit (Thermofisher Scientific, https://www.thermofisher.com). For reverse transcription PCR (RT-PCR)–positive pooled samples, viral RNA was extracted again from individual samples and checked by using conventional RT-PCR. A case of laboratory-confirmed CHIKV infection was defined as a case with positive results by RT-PCR or CHIKV IgM ELISA.

Sequencing of envelope protein 1 (E1) (402 bp) and NS1 1 (262 bp) gene regions of CHIKV was performed by using Sanger method with a 3500 Genetic Analyzer (Thermofisher Scientific). We used the maximum-likelihood method by using PHYML version 3.0.1 (http://www.atgc-montpellier.fr). Phylogenetic trees were constructed on the basis of partial nucleotides sequences of 2 gene regions for CHIKV strains. The substitution model was selected by using jmodeltest-2.1.7 (https://github.com), and generalized time reversible plus l was chosen as the model with bootstrap values after 1,000 replications. Trees were drawn by using Fig tree software version 1.4.2 (*10*). Sequences of the strains in this study were submitted to GenBank (accessions nos. MN552427–41).

Data entry was performed by using Microsoft Excel (https://www.microsoft.com), and analysis was performed by using R software version 3.4.4 (https://www.r-project.org). Our protocol was approved (Ethics/DMR/2019/082) by the Institutional Review Board of the Department of Medical Research, Ministry of Health and Sports, Myanmar.

Of 500 blood donors, 14 (2.8%, 95% C 1.7%–4.6%) were positive for CHIKV IgM, 135 (27.0%, 95% CI 23.2%–31.1%) for CHIKV IgG, 2 (0.4%, 95% CI 0.05%–1.4%) for CHIKV RNA. A total of 16 of 500 blood donors (3.2%, 95% CI 2.0%–5.1%) were confirmed as having CHIKV infection. All IgM-positive blood donors were negative for CHIKV RNA, and blood donors positive for CHIKV RNA were negative by serologic analysis. Of 151 patients with acute febrile illness, 26 (17.2%) were positive for CHIKV RNA, 4 (2.6%) for CHIKV IgM, 1 (0.7%) for CHIKV RNA and CHIKV IgM, and 9 (5.9%) for CHIKV IgG. Using these data, we confirmed that 31 (20.5%, 95% CI 14.1%–26.9%) had CHIKV infection.

**Figure 1:**
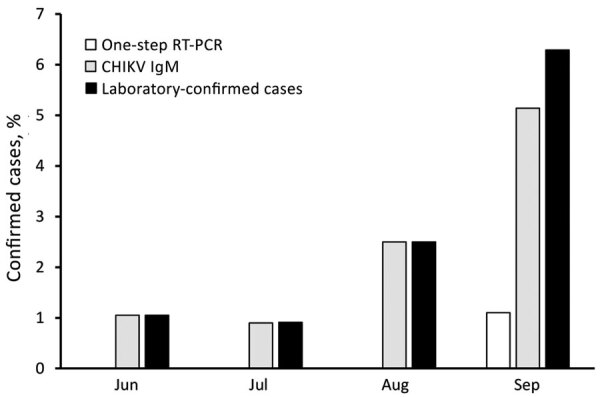
Distribution of laboratory-confirmed cases of infection with CHIKV among blood donors, Myanmar, June–September, 2019. Laboratory confirmed cases were defined as RT-PCR or CHIKV IgM positive.CHIKV, chikungunya virus; RT-PCR, reverse transcription PCR.

The proportion of confirmed CHIKV cases among blood donors had an increasing trend from June through September: prevalence rates were 1.1% in June, 0.9% in July, 2.5% in August, and 6.3% in September (p = 0.02) (Figure 1). Among infected patients, 19 had an acute viral infection and 4 had neurologic manifestations, and all blood donors were asymptomatic (Table 1).

**Table 1:** Clinical manifestations of blood donors and patients with acute febrile illness who were confirmed as having chikungunya virus infection, Myanmar

Group	No. positive/no. tested (%)
Blood donors	16/500 (3.2)
Asymptomatic	16/16 (100.0)
Patients with acute febrile illness	31/151 (20.5)
Acute viral infection	19/31 (61.3)
Meningitis	2/31 (6.5)
Viral encephalitis	2/31 (6.5)
Dengue virus infection	5/31 (16.1)
Chikungunya virus infection	1/31 (3.2)
Febrile convulsion	2/31 (6.5)


**Figure 2:**
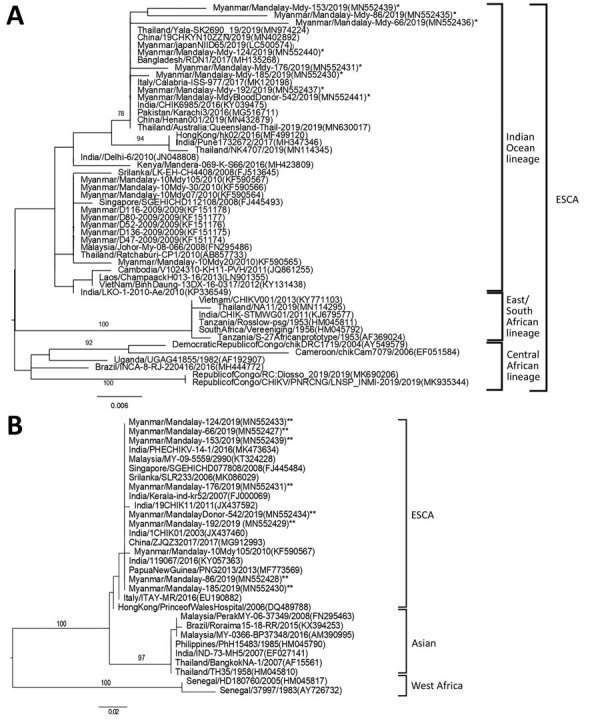
Phylogenetic trees constructed on the basis of partial nucleotide sequences of CHIKV to show the relationships of CHIKV strains from different sources, including strains detected in Myanmar during 2019 (asterisks). A) Envelope protein 1 gene; B) nonstructural protein 1 gene. Numbers along branches are bootstrap values. Representative strains of each genotype obtained from GenBank are named by country of origin, strain name, year of isolation, and GenBank accession number. Scale bars indicate nucleotide substitutions per site. ESCA, East/Central/South African.

Amino acid sequences of the partial E 1 protein of the infecting 2019 CHIKV strains in this study showed no E1:A226V mutation (Table 2). However, the E1:K211E mutation, which was not observed in strains isolated in Myanmar during 2010 having the E1:A226V mutation, was present. Phylogenetic trees for partial E1 and NS1 genes (*11*) showed that our virus strains belonged to the Indian Ocean clade of the ECSA genotype and were similar to those circulating in India and Thailand and previously circulating in Myanmar (Figure 2).

**Table 2:** Comparison of amino acids encoded by the envelope protein 1 gene of chikungunya virus strains isolated in this study with those of reference strains previously circulating in Myanmar and neighboring countries

Strain	Amino acid position																						
	208	209	210	211	212	213	214	215	216	217	218	219	220	221	222	223	224	225	226	227	228	229	230
MN552435/Mdy86/Myanmar/2019*	P	E	S	E	D	V	Y	A	N	T	Q	L	V	L	Q	R	P	A	A	G	T	V	H
MN552436/Mdy66/Myanmar/2019*	P	E	S	E	D	V	Y	A	N	T	Q	L	V	L	Q	R	P	A	A	G	T	V	H
MN532437/Mdy192/Myanmar/2019*	P	E	S	E	D	V	Y	A	N	T	Q	L	V	L	Q	R	P	A	A	G	T	V	H
MN532438/Mdy176/Myanmar/2019*	P	E	S	E	D	V	Y	A	N	T	Q	L	V	L	Q	R	P	A	A	G	T	V	H
MN552439/Mdy153/Myanmar/2019*	P	E	S	E	D	V	Y	A	N	T	Q	L	V	L	Q	R	P	A	A	G	T	V	H
MN552440/Mdy124/Myanmar/2019*	P	E	S	E	D	V	Y	A	N	T	Q	L	V	L	Q	R	P	A	A	G	T	V	H
MN552441/Mdy542/Myanmar/2019*	P	E	S	E	D	V	Y	A	N	T	Q	L	V	L	Q	R	P	A	A	G	T	V	H
KF590567/Mandalay/Myanmar/2010	P	E	S	K	D	V	Y	A	N	T	Q	L	V	L	Q	R	P	A	V	G	T	V	H
KF590565/Mandalay/Myanmar/2010	P	E	S	K	D	V	Y	A	N	T	Q	L	V	L	Q	R	P	A	V	G	T	V	H
MN114295/Thailand/2019	P	E	S	K	D	V	Y	A	N	T	Q	L	V	L	Q	R	P	A	A	G	T	V	H
MN114293/Thailand/2019	P	E	S	K	D	I	Y	A	N	T	Q	L	V	L	Q	R	P	A	V	G	T	V	H
MN114344/Thailand/2019	P	E	S	E	D	V	Y	A	N	T	Q	L	V	L	Q	R	P	A	A	G	T	V	H
MK848202/Thailand/2018	P	E	S	E	D	V	Y	A	N	T	Q	L	V	L	Q	R	P	A	A	G	T	V	H
FJ807895/Malaysia/2009	P	E	S	E	D	V	Y	A	N	T	Q	L	V	L	Q	R	P	S	A	G	T	V	H
MG516711/Karachi/Pakistan/2016	P	E	S	E	D	V	Y	A	N	T	Q	L	V	L	Q	R	P	S	A	G	T	V	H
MK473631/India/2016	P	E	S	E	D	V	Y	A	N	T	Q	L	V	L	Q	R	P	A	A	G	T	V	H
MG912993/China/2017	P	E	S	E	D	V	Y	A	N	T	Q	L	V	L	Q	R	P	A	A	G	T	V	H
MK120198/Italy/2017	P	E	S	E	D	V	Y	A	N	T	Q	L	V	L	Q	R	P	A	A	G	T	V	H

*Strains isolated in this study.

## Conclusions

We showed that 3.2% of blood donors and 20.5% of children had CHIKV infections during the CHIK outbreak in Myanmar in 2019. During 2019 in Puerto Rico, USA, 2.1% of blood donors was found to be positive with CHIKV RNA (*12*). CHIKV can be detected up to 7 days after symptom onset. IgM can appear on day 4 after symptom onset, peak at day 7, and can persist for 2–3 months. Thus, if blood donors are positive for CHIKV IgM but not viremic, there is a low risk for transfusion-transmitted infection (*13*). However, the World Health Organization recommends deferment of blood donations by persons with confirmed CHIKV infections for >6 months (*14*). To avoid risks of transfusion-transmitted infection, molecular testing for CHIKV infection must be conducted for blood donors (*11*). Detection of CHIKV IgM and IgG indicates recent (IgM) and past (IgG) infections with CHIKV. The blood donors positive for CHIKV infection in this study did not have symptoms of CHIKV infection. Thus, verbal questions about the history of infections of blood donors are not sufficient for donor screening in disease-endemic countries.

Previous studies showed that genotypes from Asia and the ECSA genotype were circulating in Southeast Asia (*11*). In Thailand, Indian Ocean and South African clades of the ECSA genotype were detected during the outbreak in 2019 (*2*). The Indian Ocean lineage of ECSA genotype has been introduced in SEA countries since 2007 (*11*). In our study, the virus strains detected in the 2019 outbreak in Myanmar belonged to Indian Ocean lineage of ECSA genotype and had close similarity to the strains circulating in 2019 in Thailand, which could be the source of CHIKV in Myanmar. The CHIKV strains seen in 2010 in Myanmar also belonged to the same clade of ECSA genotype. However, the E1:A226V mutation in these strains (*7*) was not present in the 2019 CHIKV strains. Both E1:A226V mutant and nonmutant strains were isolated in Thailand during 2019 (*2*). Furthermore, the CHIKV strains in this study had the E1: K211E mutation with an E1:226A background. A previous study reported that CHIKV strains with the E1:K211E mutation with an E1:226A background increased virus infectivity and transmission compared with nonmutant parenteral E1:226A CHIKV virus (*15*). Our study highlights the need to strengthen infection prevention control measure activities and blood donor screening during CHIK outbreaks.
